# Forensic podiatry in the identification of gait by CCTV. A systematic review

**DOI:** 10.1007/s12024-025-01022-7

**Published:** 2025-05-27

**Authors:** Aurora Castro-Méndez, Natalia Tovaruela-Carrión, Laura Regife-Fernández, Sara García-Mora, María Vázquez-Castro, Juan Alvarez-Cordero

**Affiliations:** 1 Grupo de Investigación en Liderazgo Hermes CTS-601, Departamento de Podología, Facultad de Enfermería, Fisioterapia y Podología. Liderazgo DS-30 Bases biomédicas del pie que afectan al apoyo y la marcha, Instituto de Biomedicina de Sevilla, IBiS/Universidad de Sevilla, Sevilla, 41009 España; 2 Grupo de Investigación Hermes CTS-601, Departamento de Podología, Facultad de Enfermería, Fisioterapia y Podología. Grupo de Investigación DS-30 Bases biomédicas del pie que afectan al apoyo y la marcha, Instituto de Biomedicina de Sevilla, IBiS/ Universidad de Sevilla, Sevilla, 41009 España; 3 Independent Researchers, Luis Montoto St: 91, Seville, 41018 Spain

**Keywords:** Forensic podiatry, Footprint measurement, Forensic anthropology, Gait analysis

## Abstract

Purpose: Forensic podiatry is the application of the professional knowledge of this specialist in the legal field as an expert. Forensic podiatrists collaborate in the forensic identification of evidence that, from the foot, can help clarify what happened at a crime or crime scene. At the scene of the crime, footprints, shoe prints, and traces are evidence that are present even though they might not be immediately visible. The forensic analysis of a suspect’s gait is evidence sometimes available and has as an important characteristic: it is an individualistic parameter and even a biometric factor. This gait analysis can be very relevant as evidence in the context of a crime. The objective of this systematic review is to evaluate the reliability of gait forensics with respect to the use of angular measurements compared to observational analysis of morphological characteristics to identify the current gold standard. Methods: A systematic review of the scientific literature available in PubMed, Embase, Scopus, Web of Science, CINAHL, and Dialnet has been carried out. Nine observational studies were selected after applying the eligibility criteria. Results: The selected studies were analytical and provided, among other information, numerical data on the reliability, reproducibility, and validity of the analysis methods in question. There is not enough conclusive scientific evidence on the reliability of the method of analysis using angular measurements, as there is controversy between authors. Conclusion: Forensic gait analysis based on angular measurements shows reliability limitations due to intra-individual factors (mood, clothing), requiring further empirical evidence; in contrast, the observational method analyzing of unique gait characteristics emerges as the most reliable method, offering high accuracy, validity, and reproducibility when conducted by trained biomechanical experts.

## Introduction

Forensic podiatry is a medical discipline that plays a crucial role in the identification process in legal proceedings requiring specialised knowledge in this field [1]. Crime scenes often present evidence that needs to be evaluated from a forensic podiatry perspective, such as plantar footprints (partial or complete), footwear, anatomical remains of the feet, biomechanical analyses, or images and recordings of gait captured by Closed Circuit Television (CCTV) cameras, all of which can contribute to clarifying the facts [2, 3]. Forensic podiatrists have the expertise to identify evidence related to the feet and assist in identifying individuals through their anatomical features [4, 5]. The principles and knowledge of forensic podiatry are highly beneficial for investigators at crime scenes, particularly when dealing with evidence related to feet, footwear, and/or gait. Moreover, this knowledge helps establish the physical or biological profile of a suspect for individualisation and identification [1, 3].

Forensic gait analysis has emerged as a recent specialisation within forensic podiatry, becoming increasingly recognised and utilised by experts [6]. This analysis is carried out through a structured method, which includes the phases of analysis, comparison, evaluation, verification, and the subsequent preparation of reports [3, 4]. In CCTV gait studies, the gait of a subject and its inherent characteristics are analysed and compared to contribute to the identification process [7, 8]. It is argued that each person exhibits a repetitive and distinctive gait pattern, which gives it an individualistic and recognisable quality. This pattern can allow a person to be identified from a distance by their walking style, making gait analysis a valuable tool in forensic investigations, and emerging as a biometric analysis parameter [9]. In addition to gait, footprints recovered from the crime scene, such as bare footprints, shoe prints, or sequences of footprints, also serve as critical evidence [1, 3, 10].

The use of CCTV cameras and other surveillance methods has generated growing interest in the practice and research of forensic gait analysis and its potential for human individualisation. For the first time, forensic gait analysis was used as admissible scientific evidence at the Old Bailey Central Criminal Court in London, UK, in the case of R v. Saunders in 2000 by the UK-based forensic podiatrist, Dr. Haydn Kelly [6, 8]. Since then, various countries have admitted gait analysis as a complementary form of evidence in court, although this method is still considered questionable in terms of reliability and accuracy [3, 6, 8, 11]. Similarly, gait analysis has been used in forensic practice in many countries, and camera-based computer systems have been developed to recognise a person’s highly unique movement patterns, offering distinctiveness to their gait [12]. Currently, gait recognition systems based on artificial intelligence, such as convolutional neural networks, are being implemented and have been shown to improve identification accuracy, even with low-resolution recordings [13].

Today, the majority of crimes occurring on public streets are highly likely to be captured by video surveillance cameras located in shops, banks, or other public places. CCTV images provide different views of the recorded subject (front, transverse, and sagittal, with the frontal view being the most reliable [14]), eliminating the psychological factor that might predispose an individual to simulate an unreal gait, as individuals are usually unaware they are being recorded. As a result, this method is non-intrusive and does not require the collaboration of the subject, making it particularly useful in the field of criminalistics [14, 15]. Before the invention and use of CCTV, investigative agencies relied primarily on eyewitnesses who claimed to have “seen” someone at the scene with a particular “walking style.”

In forensic gait analysis, angular measurements play a key role, as they are necessary for the analysis and the subsequent presentation of reports as evidence in legal proceedings [3, 16, 17]. Examining these angular measurements of the gait cycle is essential, as they provide quantitative and objective information about the study performed. The joint angles and those formed by various body angles have an individualistic character for each subject [18]. For an exhaustive analysis of video evidence, it is necessary to use specific software that allows tracking key points of the body and calculating joint angles during the gait cycle, such as those of the hip, knee, or ankle [18–20]. When video footage only shows a partial gait cycle (since it represents only a portion of the full cycle), evidential recognition using the observational method becomes challenging. In these cases, the use of joint measurements at specific time intervals is advocated, aiming to study the variability between suspects and obtain discriminatory parameters [21].

Therefore, gait analysis has significant potential to provide valuable evidence, as it is a unique characteristic of each individual [20]. However, improving this analysis with accurate measurements is complicated due to the low quality of some surveillance footage, which prevents a conclusive identification of a person based solely on image analysis. It cannot be definitively stated in a court of law that no other person could have a similar gait pattern based on a specific set of characteristics [3, 22]. As a result, gait analysis is not considered as strong evidence as fingerprints or DNA, but it can be useful in the absence of conclusive evidence. However, when combined with photogrammetry, gait analysis can be regarded with greater reliability [23]. Therefore, the aim of our study is to evaluate the reliability of forensic gait analysis by using angular measurements for subject identification and to compare it with observational analysis of morphological features.

## Methods

### Search strategy and inclusion criteria

A systematic review of the scientific literature was carried out in the PubMed, EMBASE, Web of Science (WoS), Scopus, Dialnet Plus, and CINAHL databases and of the gray literature from specific repositories and Google Scholar to mitigate biases. It should be noted that all the information has been collected, organized, and viewed using the Zotero bibliographic manager. The search was conducted between January and May 2024 by two independent researchers (J.A.C and M.V.C). The structure of the systematic review followed the principles proposed in the PRISMA [24]. The review protocol was registered in the Open Science Framework (OSF), under the name: “Subject identification methods in relation to forensic gait analysis using CCTV. A systematic review.” (available at https://osf.io/4byzv).

The research question followed the PICOS criteria (P-Patients: healthy adult population with normal gait; I-Intervention: Use of forensic gait analysis using angular measurements in the identification of subjects from CCTV recordings or images; C-Comparison: forensic gait analysis using observational study of individual characteristics; and O-Outcomes: Reliability of forensic gait analysis using angular measurements in the identification of subjects).

The search strategy was established using the Medical Subject Headings (MeSH) thesaurus to identify keywords. The selected terms were linked with Boolean operators (AND, OR, and NOT).

After conducting the search strategy in the different databases, duplicate articles were eliminated, and the remaining articles were reviewed by two independent reviewers for their titles and abstracts. Those that did not meet the inclusion criteria were eliminated. The following inclusion criteria were used to select the articles: articles published in the last 25 years (1999–2024), in Spanish or English, involving a healthy adult population of both sexes with normal gait, and the type of study (descriptive or observational), experimental, case reports, meta-analyses, systematic reviews, or cross-sectional studies consisting of studies in which gait analysis was performed using CCTV cameras or laboratory simulations thereof. The exclusion criteria were: articles that included subjects with a notable pathology in the gait cycle (studies of patients with neurological problems of various types, such as hemiplegia), studies that analyzed gait using advanced software or artificial intelligence, studies that excluded one sex, and studies that did not use gait analysis in the forensic field.

### Data selection and extraction

Articles retrieved with the Zotero bibliographic reference management tool were eliminated using the Check for duplicates tab. We reviewed the titles and abstracts of the articles and eliminated those that did not meet the established inclusion criteria. Regarding data extraction, the following information was obtained: author’s name, year of publication, study title, objectives, study design, sample, intervention, results, and limitations.

## Results

**Fig. 1:**
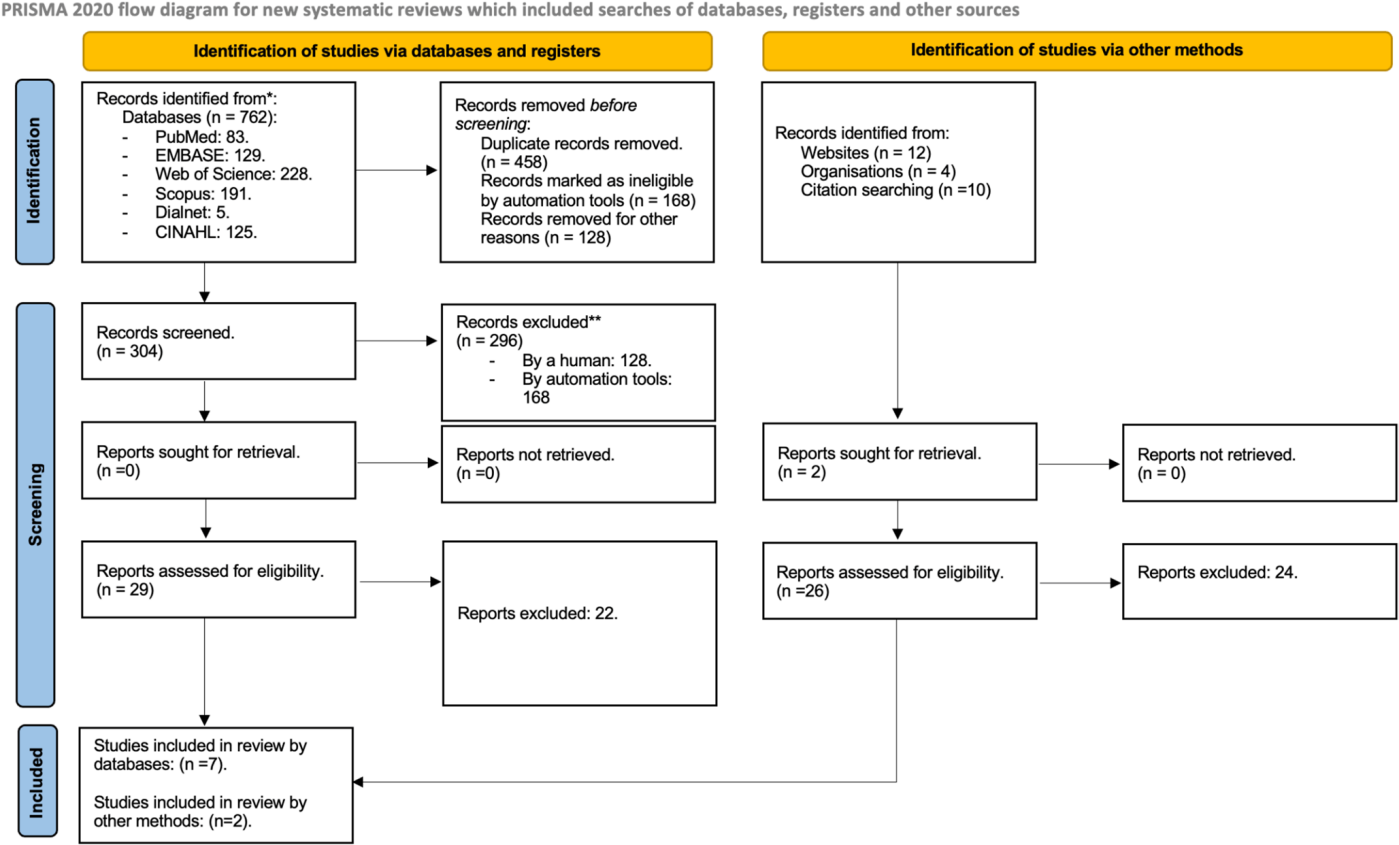
PRISMA 2020 flowchart of the selection of studies for the systematic review

From bibliographic searches, 762 articles were obtained in the six databases consulted (Table 1). After removing duplicates (458 articles), a total of 304 articles were reviewed. Following established criteria, 168 articles were discarded after reading the title and abstract. The remaining articles were read in full text, and of these, only nine were eligible after reading and meeting the inclusion and exclusion criteria (Fig. 1).

The nine articles chosen are cross-sectional descriptive observational studies. According to the Canadian Task Force of the Periodic Health Examination evidence scale, the level of evidence they acquire is III. For the assessment of the level of scientific evidence of this systematic review, the evidence quality evaluation system was used, and was officially validated and published in the Canadian Task Force on the Periodic Health Examination and in the U.S. Preventive Services Task Force [25].

Table 1 provides an individual synthesis of the objectives, methodology and results obtained from each of the studies that are part of the systematic review.

**Table 1:** Selected articles that met the inclusion and exclusion criteria of the study

*Title*,* Author and Year*	*Objective*	*Study Design*	*Intervention*	*Results*	*Limitations*
The repeatability and reproducibility of the Sheffield Features of Gait Tool. Ivan Birch et al. [27]. 2019	To check the repeatability and reproducibility of the first tool created to standardize gait forensics (Sheffield Features of Gait Tool) based on observational gait analysis tools.	Cross-sectional and analytical observational study.	Fourteen participants, each with at least 12 months of experience in observational gait analysis, watched video images of avatars randomly for 6 weeks and completed all 113 parameters of the tool for each image.	An average repeatability of 79.54% and an average reproducibility of 73.45% were obtained, demonstrating that the use of this tool by experienced analysts resulted in good repeatability and reproducibility.	Studies used computer-created avatars, not humans.
The accuracy and validity of the Sheffield Features of Gait Tool. Ivan Birch et al. [28]. 2021	Check the accuracy of the evaluators, and therefore, the validity of the gait forensic analysis tool.	Cross-sectional and analytical observational study.	Comparison of the gait parameters assessed by each of the fourteen participants in each imaging study against the correct parameters previously programmed in each avatar.	The analysts achieved an accuracy of 74.96% in identifying the gait exhibited by the avatars.	Studies used computer-created avatars, not humans.
Identification of individuals by observational gait analysis using closed-circuit television footage. Louis Raymond et al. [29]. 2013	To verify the effectiveness of observational gait analysis as a means of identification, this study investigated the capacity of experienced analysts (minimum 5 years).	Cross-sectional and analytical observational study.	Seven experienced analysts each viewed 5 CCTV footage recorded with the same camera and perspective, each showing a target walker and five suspects in identical clothing.	The final result showed that the analysts were able to correctly discriminate whether the suspect’s gait was compatible with the target’s in 71% of cases. This was compared to a previous study of observational analysis by non-experts, concluding that experienced analysts were 21% more successful.	Small sample of experienced podiatrists.
Visual Analysis of Gait as a Cue to Identity Sarah V. Stevenage et al. [30]. 1999	To determine whether walking can serve as a reliable indicator of identity through observational analysis by inexperienced students.	Analytical cross-sectional observational study	First, the number of attempts to identify a subject in a mixed design with different lighting conditions was assessed as a dependent variable. Finally, the number of correct identifications and the confidence in selection in a design with variable conditions were assessed as dependent variables.	The human visual system was shown to be sophisticated enough to learn to identify six individuals based on their gait in simulated daylight, simulated dusk, and spotlight displays. Even under adverse viewing conditions involving a single brief exposure, humans could identify a target in a “walking identity parade” at levels higher than chance.	Age of study
The identification of individuals by observational gait analysis using closed circuit television footage: Comparing the ability and confidence of experienced and nonexperienced analysts María Birch et al. [18]. 2020	To compare the skills and confidence of participants with experience in observational gait analysis and inexperienced participants using the methodology of Louis Raymond et al. 2013.	Cross-sectional and analytical observational study.	The participants were divided into two groups: 11 professionals in gait analysis (podiatrists) and 19 non-professionals in this field. The methodology of the study by Louis Raymond et al. 2013 was used, and, in addition to identifying the target and suspicious subject, the participants had to mark their level of confidence in their decision using a scale.	The results showed a higher rate of correct identification and a higher level of confidence in observational gait studies conducted by experienced analysts compared to non-experienced ones. In conclusion, the study highlighted the importance of training and experience to improve the effectiveness of gait analysis in CCTV images.	Poor quality of the video studied.
Use of gait parameters of persons in video surveillance systems. Zeno Geradts et al. [16]. 2002	Compare and evaluate the usefulness of using measurements of different gait parameters to identify people through video surveillance systems.	Cross-sectional and analytical observational study.	Eleven healthy individuals with an average age of 23.2 years and an average body mass of 73.5 kg had markers placed on different joints and wore the same clothing. Participants were asked to walk at their usual speed along a camera path with different perspectives. Seven trials were conducted for each subject.	The parameters that showed a significant difference between subjects were the angle of the foot, the angle of the hip joint and stride length. The other parameters obtained a score of less than 25% which means they are poor for recognition purposes	The actual situation differs from the experimental situation in which the study is conducted. (clothing, footwear, camera location, speed).
Variability and Similarity of Gait as Evaluated by Joint Angles: Implications for Forensic Gait Analysis Sylvia Yang et al. [18]. 2014.	To analyse the discriminating power of joint angles throughout a gait cycle using CCTV.	Cross-sectional and analytical observational study.	Walking data was collected from 12 healthy men using 5 VGA video cameras while they walked barefoot. Reflective markers were placed on 15 different joint segments of the lower limb.	The discriminating power and variability of the joint angles recorded at a given time and plane of the gait cycle can be evaluated. This tool better supports statements in forensic gait cases about whether a suspect and perpetrator could be the same person.	This gait cycle assessment method does not require the recording or study of a full cycle.
Gait Recognition Using Joint Moments, Joint Angles, and Segment Angles P.K. Larsen. [22]. 2010	To examine the ability to recognize individual joint moments, joint angles, and segment angles in the three planes using CCTV recorded images under laboratory conditions.	Cross-sectional and analytical observational study	Six healthy women and fifteen men, with an average age, height and weight of 34 years, 170 cm, and 61 kg, respectively, were recruited for the study. Fifteen sherical markers were placed on anatomical landmarks on the lower extremity, and a left-right gait cycle was recorded for each subject in all tests.	High recognition rates have been found, especially when analysing joint and segment angles in the sagittal and frontal planes using correlation analysis. The variables in the frontal plane showed greater interindividual differences.	Choice of a fixed speed.
Intra-individual gait pattern variability in specific situations: Implications for forensic gait analysis Oliver Ludwig et al. [20]. 2015.	To examine inter-intra-individual differences in gait patterns in different gait situations using phase diagrams of lower extremity angles in order to draw conclusions about the suitability of gait pattern analysis for identification.	Cross-sectional and analytical observational study.	Under biomechanics laboratory conditions, subjects (5 men and 3 women) walked a 6-meter-long walkway at a given speed. This was recorded with a camera positioned 5 m away and 1.5 m high. Five variants were recorded for all subjects: barefoot, sports shoes, full-face helmet, combat boots, and fatigue. Each subtest was repeated 3 times.	The study showed the adaptability of the gait pattern, which varies under different conditions. This variability complicates the comparison between a perpetrator recorded by CCTV and the suspect’s comparative recordings. Gait patterns change depending on multiple factors. While it is difficult to definitively identify a subject for the reasons mentioned, gait analysis could be quite useful to exclude individuals from a pool of possible suspects.	High similarity in intra-individual measurements

## Discussion

Forensic podiatry has emerged as a vital discipline within criminalistics, playing an increasingly important role in legal proceedings [5]. This specialty focuses on applying podiatric knowledge to provide scientific evidence that can be used in court, particularly in identifying individuals through podological clues, such as footprints, footwear, and gait analysis [1]. Within this field, forensic gait analysis has become central, with methods like angular measurements being widely utilised for identifying individuals based on gait patterns. This research aimed to examine the reliability of these methods by comparing angular measurement analysis with the traditional gold standard of observational studies.

Gait patterns are considered valuable forensic evidence due to their kinematic characteristics, which can be unique to each individual, making them a potential tool for discrimination in criminal investigations [26, 27]. Birch et al. [26] assert that gait analysis, particularly the examination of walking style, is a crucial part of forensic science, with the method gaining prominence as a biometric tool for individualisation. The first forensic use of gait analysis in court occurred in 1839, with significant advancements since then, including automated systems that recognise gait patterns [27]. Larsen et al. [22] highlighted that, although more empirical research is needed, gait analysis offers an advantage over other biometric methods, such as facial recognition, which can be compromised if the perpetrator attempts to conceal their face.

Among the popular forensic gait analysis methods, angular measurements have shown promise, focusing on joint angles and ranges of motion during gait cycles [17, 28, 29]. However, recent studies by Birch et al. [17, 28] underscore the effectiveness of observational gait analysis from CCTV recordings, demonstrating its high applicability, repeatability, and reproducibility. While angular analysis is reliable in controlled environments, its accuracy can be influenced by factors such as clothing or footwear, and variability in joint measurement placement [19]. Larsen et al. [22] suggest that incorporating additional variables from multiple planes of movement could improve gait analysis, contributing to higher recognition rates.

Further contributions by S. Yang et al. [21] focused on enhancing the reliability of angular analysis, especially when incomplete gait cycles are captured by surveillance cameras. Their research demonstrated that focusing on specific points within the gait cycle with higher discriminatory power can improve the identification process. Nevertheless, the study by O. Ludwig et al. [19] also highlighted that various factors, including environmental conditions or the type of footwear, could affect the reproducibility of angular measurements, thus limiting their effectiveness.

On the other hand, observational gait analysis, relying on biometric recognition through CCTV footage, is considered more flexible in real-world conditions where other markers may be obscured or hidden. According to Birch et al. [17] and Raymond et al. [30], this non-intrusive method offers distinct advantages in law enforcement and security applications. Their research indicates that experienced analysts are significantly more accurate in identifying individuals based on gait analysis, showing that familiarity with the process improves recognition rates [17]. Additionally, the “Sheffield Features of Gait Tool,” developed by Birch et al. [26], has been widely tested for repeatability and reproducibility, showing promising results in the evaluation of gait from CCTV footage.

Although the reliability of the “Sheffield Features of Gait Tool” has been established, challenges remain regarding the standardisation of forensic gait analysis methods worldwide. The adoption of a universal protocol, as seen in regions like the UK, Denmark, and the US, is crucial for enhancing the reproducibility and credibility of forensic podiatry [26]. Despite some limitations identified in the studies, such as the influence of various external factors on gait patterns, observational analysis through CCTV remains the most reliable method for forensic gait analysis, particularly when combined with advanced tools like the Sheffield Features of Gait Tool.

This systematic review is not without its limitations, which should be considered when interpreting the findings. One significant limitation is the lack of scientific articles that directly compare the reliability of various methods for forensic gait analysis. This gap in the literature makes it difficult to establish a clear of methods and determine which approach is most effective in forensic applications. Furthermore, the majority of the studies included in this review are observational in nature, and the search did not yield studies with higher levels of scientific evidence, such as randomized controlled trials, cohort studies, or case-control studies, which could have provided more robust and generalizable results. Additionally, there is a noticeable lack of studies on forensic gait analysis conducted in Spain, with all the methods analyzed originating from foreign authors, many of whom have implemented their approaches in countries other than Spain. This geographical gap limits the applicability of the findings to Spanish forensic contexts. Lastly, the field of forensic gait analysis has been developed by a relatively small group of authors, with many of the articles reviewed originating from the same researchers, which may restrict the diversity of perspectives and innovations in the field. These limitations underscore the need for further research, including comparative studies, higher-quality evidence, and a broader geographical and authorial scope, to strengthen the field and ensure its broader applicability in forensic science.

## Conclusions

Regarding the reliability of forensic gait analysis using angular measurements, it is concluded that more empirical information is needed, since, although some authors affirm levels of positive correlation in the combination of angular variables in the different planes, there are authors who state that the change in intra-individual conditions (mood, clothing) make identification impossible by this method.

Regarding the comparison between the method of analysis by means of angular measurements and the method of observational analysis of individual characteristics, we can conclude that the latter is imposed as the method with the greatest scientific evidence as it has high levels of precision, validity and repeatability when practiced by professionals in the biomechanical study of gait and by overcoming the limitations expressed by the authors in terms of the analysis of angular measurements.

## Key points

1. Gait forensic analysis identifies subjects through gait characteristics and comparison.
2. Gait forensics is vital when biometric data like fingerprints or face are unavailable.
3. Studies show that angular variables in gait offer high inter-individual variability and discriminatory power.
4. The gait cycle analysis tool “Sheffield Features of Gait” demonstrates high accuracy, validity, and repeatability in subject identification.

## Abbreviations

CCTV: Closed Circuit Television
DNA: Deoxyribonucleic Acid
PRISMA: Preferred Reporting Items for Systematic Reviews and Meta-Analyses
WOS: Web of Science
CINAHL: Cumulative Index to Nursing and Allied Health Literature
OSF: Open Science Framework
MeSH: Medical Subject Headings
